# The impact of COVID-19 on the undergraduate medical curriculum

**DOI:** 10.1080/10872981.2020.1764740

**Published:** 2020-05-13

**Authors:** Preeti Sandhu, Maisie de Wolf

**Affiliations:** Faculty of Life Sciences and Medicine, King’s College London School of Medical Education, London, UK

**Keywords:** Medical education, education, medical curricula, online teaching, undergraduate

## Abstract

The coronavirus pandemic has impacted medical education globally. As universities seek to deliver medical education through new methods of modalities, this continuing of education ensures the learning of the future workforce of the NHS. Novel ways of online teaching should be considered in new medical curricula development, as well as methods of delivering practical skills for medical students online.

The coronavirus disease (COVID-19) pandemic has had a worldwide impact on the population, global economy and heath care systems. Whilst the spread of the virus has resulted in far-reaching consequences, the closure of schools and universities has led to innovative methods of delivering education, ensuring that students continue to receive teaching, albeit different methods of modality. A national effort by UK medical schools in graduating early nearly 5,500 final-year students will allow for these interim Foundation Year doctors to support the NHS during the pandemic [1]. As medical students in our penultimate clinical year of undergraduate education, we have experienced cancellations of all clinical placements into the beginning of final year, with online learning presently proving essential in the continuation of medical education.

The coronavirus pandemic has seen the introduction of novel methods of delivering education to medical students. Lectures have rapidly been developed to be delivered online as webinars using various platforms such as Zoom, with such technologically enhanced approaches already being proven to have high levels of engagement with medical students [2]. With international students making up 19.6% of the total student population [3], and many having returned to their native homes during the coronavirus outbreak, online teaching platforms are beneficial due to their worldwide accessibility, ensuring that all medical students regardless of their current location are able to access webinars as they happen or can be recorded for later use. Thus far, our experience of online webinars that have included key clinical conditions, case studies and examination questions have been well received, with a regular number of medical student attendees engaging in these lessons throughout these unprecedented times.

The transition to online medical education has also seen a change in examination methods. Following on from the recent success of Imperial College London’s first ever online exam for final years [4], other medical schools are also adopting a similar approach to ensure students remain engaged with their studies, with many universities adopting an open-book examination (OBE) approach. The introduction of OBEs will be a sudden change to nearly all medical students from previous exam-hall settings. However, OBEs have been shown to reduce student anxiety [5] and with a global level of heightened fear and apprehension during the current COVID-19 pandemic, an approach to examining students during this difficult time that can minimise symptoms of stress is welcomed.

To ensure the future workforce of the NHS are qualified, continuing education is vital and this can be achieved by medical school staff continuing to engage regularly with medical students using online teaching platforms. The current success of online teaching and OBEs provides an initial insight into new and innovative ways of teaching for medical education. Consideration is encouraged as to how such online methods may be adapted to deliver teaching on clinical and practical skills remotely that would otherwise have been developed during clinical placements.
